# Supplementary figures and images for: Is there a fair allocation of healthcare research funds by the European Union?
Source: PLoS One. 2019 Apr 15;14(4):e0207046. doi: 10.1371/journal.pone.0207046 (PMC6464186; doi:10.1371/journal.pone.0207046)

S1 Fig. Correlation plot between GDP per capita and DALY


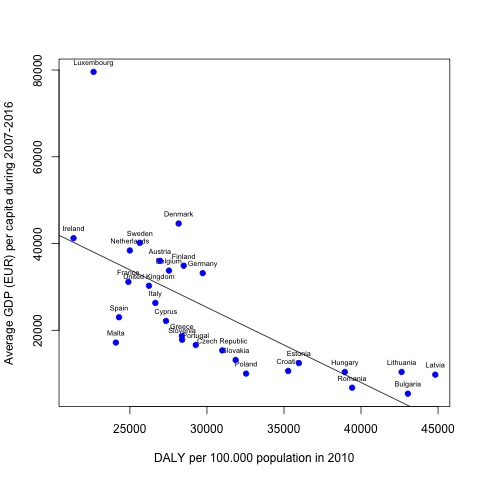

Supplement: S1 Fig — (DOCX) [file pone.0207046.s001.docx]
