# Supplementary material for: Is there a fair allocation of healthcare research funds by the European Union?
Source: PLoS One. 2019 Apr 15;14(4):e0207046. doi: 10.1371/journal.pone.0207046 (PMC6464186; doi:10.1371/journal.pone.0207046)

S1 Files. Scatterplots of the univariate analysis

Figure A: Relationship between GDP and FP7/H2020 health grants between 2007 and 2016


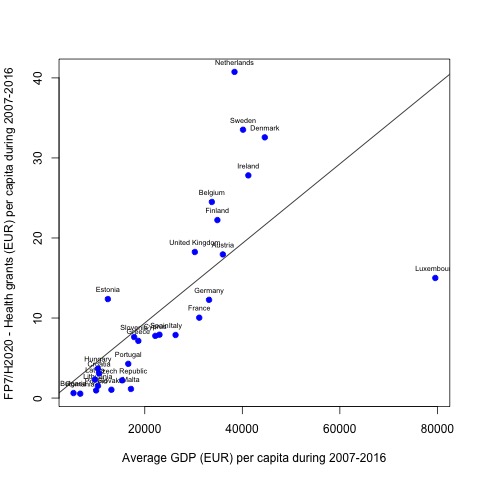


Figure B. Relationship between the disease burden and FP7/H2020 health grants between 2007 and 2016


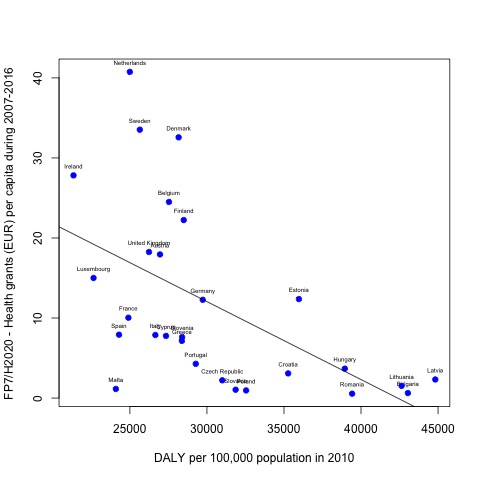


Figure C. Relationship between medical research excellence and the FP7/H2020 health grants between 2007 and 2016


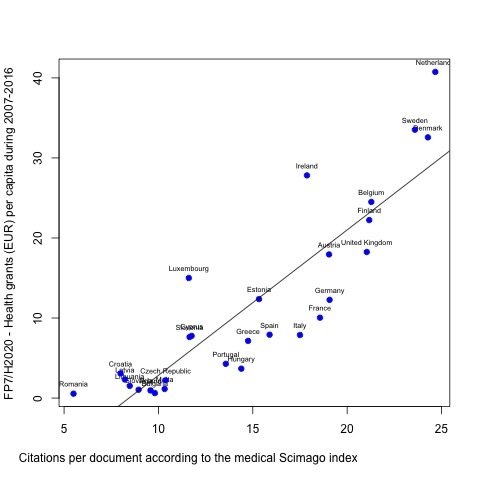


Figure D. Relationship between population size and the FP7/H2020 health grants between 2007 and 2016


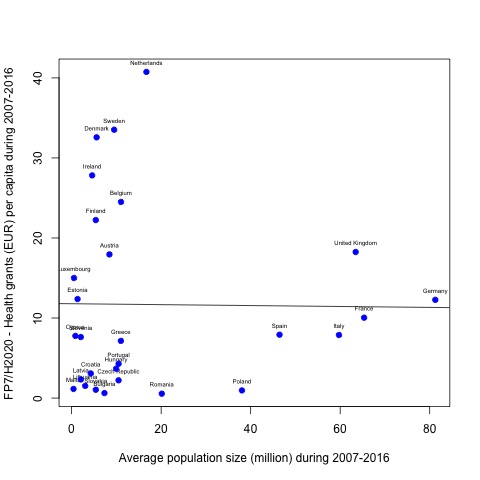

Supplement: S1 File — (DOCX) [file pone.0207046.s002.docx]
