# Supplementary material for: Is there a fair allocation of healthcare research funds by the European Union?
Source: PLoS One. 2019 Apr 15;14(4):e0207046. doi: 10.1371/journal.pone.0207046 (PMC6464186; doi:10.1371/journal.pone.0207046)
Supplement: S1 Table — (DOCX) [file pone.0207046.s003.docx]

S1 Table. Explanatory variables for the regression analyses: An overview of the country-specific explanatory variables that were included in the regression analyses

| Country | Average GDP per capita between 2007 and 2016 | Average population size between 2007 and 2016 | DALY per 100,000 inhabitants in 2010 | Citations per document according to the Scimago medicine index between 1996 and 2016 |
| --- | --- | --- | --- | --- |
| Austria | 36,011.11 | 8,444,778 | 26,953.34 | 22.69 |
| Belgium | 33,755.56 | 11,027,357 | 27,536.37 | 27.13 |
| Bulgaria | 5,388.89 | 7,332,195 | 43,042.57 | 10.60 |
| Croatia | 10,622.22 | 4,268,376 | 35,271.56 | 8.60 |
| Cyprus | 22,155.56 | 834,819 | 27,342.76 | 14.76 |
| Czech Republic | 15,366.67 | 10,482,681 | 30,995.82 | 12.25 |
| Denmark | 44,611.11 | 5,584,439 | 28,163.01 | 28.05 |
| Estonia | 12,433.33 | 1,325,462 | 35,969.32 | 24.96 |
| Finland | 34,888.89 | 5,399,086 | 28,493.84 | 32.25 |
| France | 31,166.67 | 65,340,284 | 24,901.83 | 20.71 |
| Germany | 33,177.78 | 81,248,538 | 29,727.28 | 20.78 |
| Greece | 18,677.78 | 11,006,329 | 28,375.28 | 17.71 |
| Hungary | 10,388.89 | 9,942,285 | 38,949.03 | 18.22 |
| Ireland | 41,233.33 | 4,581,373 | 21,340.48 | 21.96 |
| Italy | 26,311.11 | 59,725,729 | 26,651.29 | 21.13 |
| Latvia | 9,766.67 | 2,063,329 | 44,820.42 | 25.44 |
| Lithuania | 10,400.00 | 3,035,540 | 42,635.81 | 18.17 |
| Luxembourg | 79,566.67 | 526,887 | 22,641.20 | 18.24 |
| Malta | 17,166.67 | 419,535 | 24,089.55 | 15.59 |
| Netherlands | 38,400.00 | 16,704,559 | 24,996.27 | 28.97 |
| Poland | 10,022.22 | 38,050,457 | 32,541.11 | 11.27 |
| Portugal | 16,611.11 | 10,492 855 | 29,275.34 | 15.95 |
| Romania | 6,766.67 | 20,140,426 | 39,421.26 | 8.97 |
| Slovakia | 13,155.56 | 5,402,225 | 31,862.30 | 12.10 |
| Slovenia | 17,822.22 | 2,049,140 | 28,862.30 | 15.00 |
| Spain | 23,011.11 | 46,445,553 | 24,294.30 | 16.02 |
| Sweden | 40,144.44 | 9,497,501 | 25,643.59 | 29.08 |
| United Kingdom | 30,266.67 | 63,461,799 | 26,239.13 | 24.98 |
