# Supplementary material for: Is there a fair allocation of healthcare research funds by the European Union?
Source: PLoS One. 2019 Apr 15;14(4):e0207046. doi: 10.1371/journal.pone.0207046 (PMC6464186; doi:10.1371/journal.pone.0207046)
Supplement: S2 Table — (DOCX) [file pone.0207046.s004.docx]

S2 Table. Results from the initial multiple regression model

| Variable | Coefficient | P-value | Overall P-value | 95% Confidence interval | R^2^ |
| --- | --- | --- | --- | --- | --- |
| GDP per capita (1000 EUR) | 18.41 | 0.097 | - | -3.64; 40.46 | 0.83 |
| DALY | -30.15 | 0.222 | - | -79.93; 19.64 |  |
| Research excellence | 100,283 | < 0.001 | - | 60,269; 140,298 |  |
| Population (2nd quartile) | 673,962 | 0.031 | 0.082 | 69,270; 1,278,655 |  |
| Population (3rd quartile) | 633,305 | 0.048 |  | 6,829; 1,259,782 |  |
| Population (4th quartile) | 190,753 | 0.534 |  | -436,460; 1,352,454 |  |

GDP: gross domestic product; DALY: disability adjusted life years
